# Stress–Strain Field in an Innovative Metallic Dam Gate Used to Control the Water Flow

**DOI:** 10.3390/ma15072689

**Published:** 2022-04-06

**Authors:** Calin Itu, Sorin Vlase

**Affiliations:** 1 Department of Mechanical Engineering, Transilvania University of Brasov, B-dul Eroilor, 20, 500036 Brașov, Romania; calin.itu@unitbv.ro; 2 Romanian Academy of Technical Sciences, Bul. Dacia 26, 030167 Bucharest, Romania

**Keywords:** dam gate, metallic skin, break, structure, water flow

## Abstract

The paper aims to determine the stress and strain field in metallic dam gates to identify an optimal constructive solution for their design, from the point of view of strength in service. The study is of a dam with a central, oscillating pivot, which has the role of closing the gates when the downstream water level becomes too high and can thus flood the upstream portion of the river. It starts from a constructive solution initially proposed by the designers, which is then modified in several steps, until a better solution is reached in terms of strength to mechanical stress. This solution is obtained after analyzing several structural scenarios. The final results ensure an excellent behavior of the mechanical stresses, and represent a constructive solution that is easy to achieve and is economically convenient.

## 1. Introduction

A dam gate represents a barrier to stop or restrict the flow of water. Reservoirs controlled by dam gates offer water for a wide range of activities, such as navigability, irrigation and agriculture, household consumption, industry, hydropower, and aquaculture. Dams serve to retain water, while floodgates or levees (so called dikes) are used to manage the use of this water. With the help of dams, the possibility of creating water accumulation on the respective river is created. In conclusion, dams are used to control and stabilize water flow. To realize this, dam gates are commonly used.

The role of this mobile gate is to maintain a constant level in the free level adductions, in order to protect them from variations in the downstream water level. A number of interdependent factors must be taken into account in their design and establishment, which are the subject of technical-economic, environmental, and political analyses. These factors refer to the hydrological aspects (flows), hydraulic (flow regime, speeds, and alluvium transport), geological (foundation conditions or foundation improvement works), topographic (optimization of available space, positioning of the structure, access to the site during construction), environmental (minimizing environmental impact), and aesthetics. Mobile dams are made up of two parts: the fixed part and the mobile part. The paper will study the field of stress in the moving part of the gate. The fixed part of the dam is made of monolithic elements or structural units, connected to each other by joints and acting as a whole as a continuous structure. The source of discontinuities in a solid concrete structure is the conditions of pouring the concrete, as well as the flexibility requirements of the structure.

A major problem in the operation of these assemblies is dam-break. A number of papers address this central issue of the field. For example, a solution of the dam-break problem with partial uplift of the sluice-gate is described in [1]. This solution is obtained under the classical hypothesis of the mechanics (energy–momentum (E-M) formulation). The flow characteristics at the gate are used. The risk of breaking the dam is one of the main problems of study, and its results help decide the feasibility of building such a construction. Experimental research of the dam rupture flow through a single oblique obstruction in a right rectangular channel is presented in [2]. The breaking rate of the dam is determined based on the height of the water in the tank. If we compare these results with the previously obtained results [3], where the breaking mechanism of the dam is made with a lifting gate, there are some differences regarding the magnitude of the phenomenon, which requires future research.

Dam-break flows are studied, in experimental tests, by a moving gate that will release a large quantity of water almost instantaneously. There are two main strategies to obtain these flows: a downward moving gate or an upward moving gate. A study of the results obtained using a downward moving gate model is presented in the literature [4]. The results offer an understanding of the initial stages of the dam-break flow. Another problem that occurs in the case of dams is the breaking wave of the dam, which manifests itself as a kind of broken wave, and can affect structures and constructions downstream. It is another problem studied in the case of numerous works. A numerical model for solving this problem is presented in the literature [5]. The breaking wave is generated by the sudden lifting of the lock or the rapid opening of the gates. In this way, the water in the tank moves, in large quantities, downstream and can cause significant damage. The history of downstream water pressure initially shows a significant peak, then a fluctuating value and finally a quasi-stationary value.

For the study of the breaking wave, elaborate and well-studied analytical methods are developed (taking into account the negative impact that these phenomena can have). The problem in practice is to have models that are simple enough to be applied to practical cases by designers. A smoothed particle hydrodynamics (SPH) model that meets the above requirements is developed in [6]. The purpose for which the model is used is to study the influence of the gate opening on the wave propagation characteristics. The studied model is experimentally validated in the laboratory using employed swing and lift gates. There are theoretical solutions for obtaining a perfect breaking wave, but never, due to technical and economic limitations, can this be achieved in practice. The influence of different factors on the breaking wave is studied.

Other works have dealt with the development of numerical methods that allow for the calculation and control of break waves. The Navier–Stokes equations are solved with the limit condition [7]. The law of movement of the gate is imposed as the input date. The result is that the movement of the gate has a significant influence on the process of water flow. In a correct model, it is absolutely necessary that this movement be taken into account. The influence of a swing gate on the generation of dam-break waves is also studied. This is because this type of gate is more economical and easier to design and build [8]. The opening time of the gate is proven to have a linear dependence on the arrival time of the wave, which is important for designers to know. Some examples of solving the problems related to the construction and functions of dams are presented in [9,10,11]. Dam solutions and projects are described for real situations, for dams of great economic importance.

Another study direction is to estimate the effects of dams and the accumulation of water on the environment [12,13,14]. This research direction is not the subject of our research.

The dynamic stresses and vibrations that can occur in such a structure have also been the subject of studies. For example, in [15], the vibrations in the mechanical elements of the dam are analyzed. They can cause damage to the elastic structure and, as such, require serious design attention. The structural response to the vibrations caused by the flow is influenced by the mass and elastic characteristics of the structure, but also by the hydraulic parameters. The mentioned work analyzes the vibrations that appear in the sliding gates of the dam. It is considered that the real model of the gate is a self-excited model. Theoretical results are sustained and improved with measurements made on a real structure. In this way, models are obtained that can be useful to designers.

Numerical models for the calculation methods established in the field are studied in [16,17]. The numerical methods used are decisively based on the Finite Element Method (FEM) [18,19,20] to study the hydraulic forces, the flow parameters in the pipe, and the natural frequencies of the radial gate. The research results are applied for concrete situations, as encountered in practice. The calculations performed during the works show a good concordance of the model with the experimental results for the distribution of the pressure on the flow field and on the gate. Other problems concerning the dam break are presented in [21,22,23,24,25], and an analysis of risk management of such structures is presented in [26].

There are many dam construction solutions. In our case, we have a mobile dam, in which the two gates have a rotational movement around a central tilting pivot. On this given constructive variant, with the gate closing solution established, a calculation will be made to determine the stress that appears in the dam gates, using FEM (a method that has proved its usefulness and accuracy). Several constructive scenarios will be analyzed, starting from a solution proposed by the designers. Finally, a variant of the structure of beams covered with a metallic skin is obtained. The solution offers a good strength and, at the same time, represents a convenient economic solution.

## 2. Description of the Dam Gates

There are many dam construction solutions. In our case, we will have a mobile dam, in which the two gates can have a rotational movement around a central tilting pivot. The two gates are connected by cylindrical joints to a central shaft, which can tilt and, through this, can bring the gates into the closed position. 

The fixed concrete structure is completed by the overflow sill, which also ensures the sealing at the bottom of the dam. The sill is a massive concrete construction whose height can be close to or well above the level of the riverbed. The main function from a structural point of view is the support for dams, cofferdams, foundation for piles, and abutments in certain composition configurations. The main parameters on which the adoption of the design solution depends are the dimensions of the watercourse, site configuration, hydraulic conditions, space available on site, access possibilities, the presence of a prefabrication plant in the area, and navigation conditions (locks, bridges, and depth of navigation).

**Figure 1:**
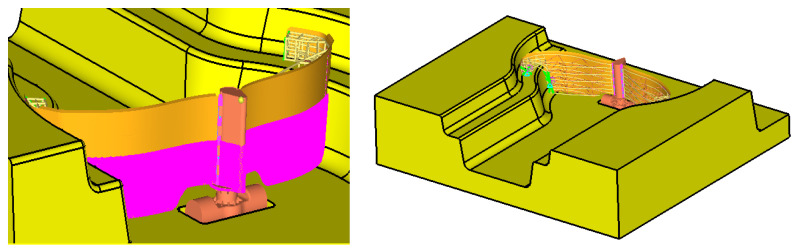
The position of gates when closed.

Figure 1 presents a dam with mobile gates, in a situation when the gates are closed. In the left part of the gates, there is also a level of water. 

**Figure 2:**
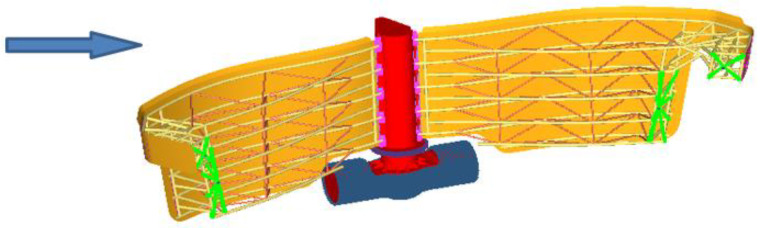
The mechanism that assure the mobility of the gates.

The case of closed gates (where the state of stress and strain for the gate structure will be calculated) is shown in Figure 1. The gates are reinforced with a beam structure covered with a metallic skin. This surface is compact and does not allow water to pass to the other side. Water accumulates on the left side of the dam, but on the right side there is also a water level that determines the operation of the closure system. After reaching a certain level of water on the right side of the dam, downstream, the dam closes and blocks the passage of water. The closure system is presented in Figure 2. It is considered that the dam will operate according to the design tasks, i.e., it will close when the downstream water exceeds a certain height. At this point, the gates close and the upstream water will accumulate inside the dam until it opens again. The main task of such a type of mobile dam is to stop the downstream water from entering the river water drain and flooding upstream portions of land.

The loads acting on the dams and that where considered in the FEM model are as follows:

- own weight, including the weight of the piles, screed, dams, bridge, and other mechanical equipment;
- horizontal and vertical hydrostatic pressure;
- static and dynamic under-pressure;
- ice pressure (if applicable);
- the pressure of the alluvium;
- wave pressure;
- earthquake loads—inertial component and hydrodynamic component;
- loads due to temperature variations;
- wind action;
- forces caused by traction or braking on the tracks.

**Figure 3:**
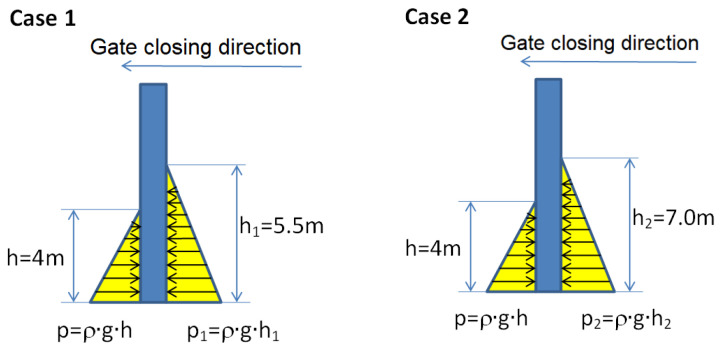
The two cases considered to compute the stress field.

**Figure 4:**
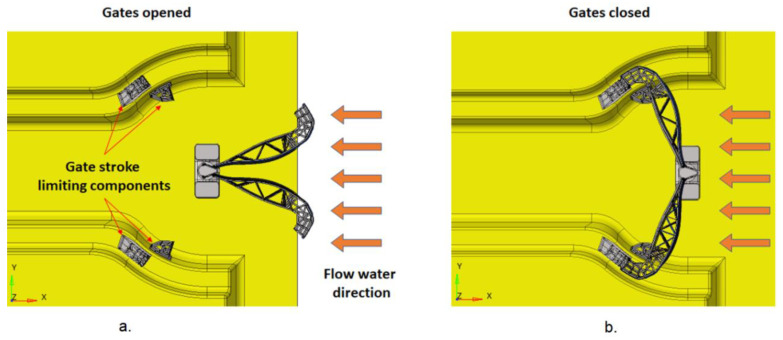
View of: (**a**) open gatesand (**b**) closed gates.

Figure 3 presents the load due to the pressure of the water on the wall of gates and Figure 4 presents a view of the open gates and closed gates.

## 3. FEA Model Used to Analyze Different Load Design Scenarios

FEM was used to determine the stresses that occur in the structure of the dam. A static analysis of the structure was performed in the paper. In the first stage, a calculation was performed to see the behavior of the elements of the structure for a constructive solution proposed by the designers. It was found that the stresses that occurred within the structure exceeded the limits accepted for the strength of the materials. Consequently, it became necessary to seek solutions to bring these stresses within acceptable limits. In this breakthrough, several scenarios were studied, as proposed by the designers, in order to reduce the load of the elements of the structure. The aim was also not to complicate the construction of the structure and not to increase the costs related to the construction and maintenance of the gate elements. 

**Figure 5:**
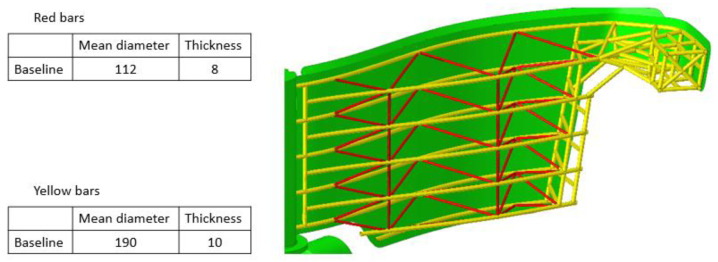
The structures of the reinforced beams used.

Figure 5 presents the FEM model of the gates [26,27,28,29,30,31]. In front of the gates there is a metallic skin assuring the impermeability of the gates to water. This skin has a low rigidity and, as a consequence, was reinforced by a beam structure. This solution is simple and easy to model. The gates were modeled with shell type elements (planar element used for situations when one size is much smaller than the other two). All bars that carry out the reinforcement behind the gates have been modeled with beam type elements.

The type of finite element used in the study based on numerical methods on the structure made in Altair Hyperworks has four corner nodes, being shell elements. The calculation of the stiffness matrix of this element will be performed starting from the general equations of the theory of elasticity of the isotropic medium. This shell element has four corner nodes, six degrees of freedom per node (three translations and three rotations). The constitutive law used in the commercial software is the one described in [32] or [33] for such types of elements.

In all of the scenarios, the thickness and implicitly of the section were not changed from one analysis iteration to another, but were only suggested as a method to improve the reinforcement. Below are the data that define the cross section of the beams that form the structure of the lattice beams.

The structure of the lattice beams and the plate are welded to the structure of the bars.

Scenario 1. Initial proposed solution. 

A first calculation of the stress in the structure of the gates is made on the solution presented in the first scenario. The middle pillar was modeled with solid tetrahedral elements. The hinges connecting the gate and the pillar were modeled with beam type elements (one-dimensional bar element) with a degree of rotational freedom. The water pressure acts on the inside and outside of the gates. The static pressure was applied based on the following relation:

(1) p=ρgh,

ρ is water density (1000 kg/m^3^), *g* is gravity acceleration (9.81 m/s^2^), and *h* is the height of the water level considered.

In the following are the detailed boundary conditions. Two load cases are considered in which the heights of the water level on the two sides of the gate are different. Based on these water levels, the hydrostatic pressures are calculated. The water pressure at the base of the dam upstream is p=0.039 MPa and downstream is $p_{1}=0.054\;\mathrm{MPa}$, $p_{2}=0.069\;\mathrm{MPa}$.

**Figure 6:**
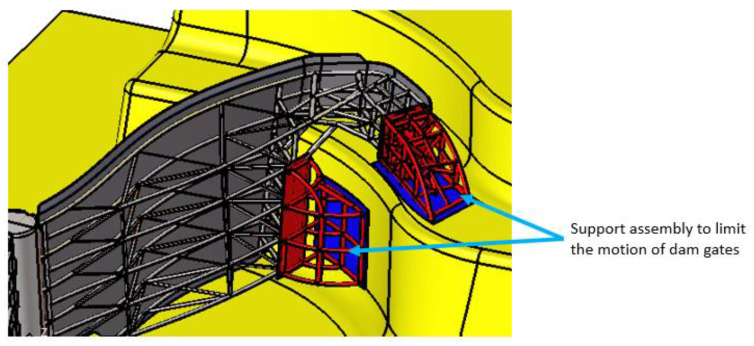
Support assembly.

**Figure 7:**
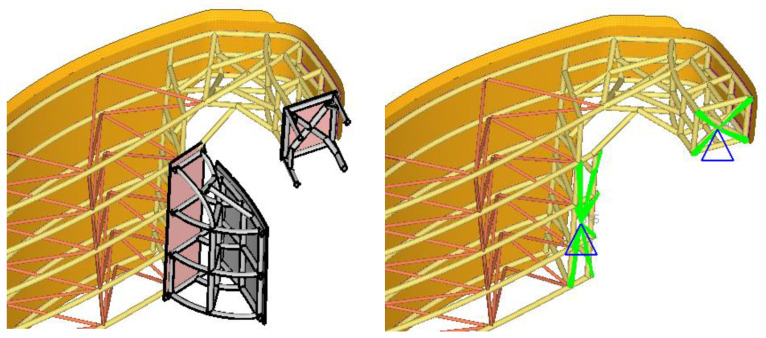
The boundary conditions presented for the gates.

The gates, the pivot, and the structural beam are meshed with different types of finite elements. In the stop areas, in the FEM model, the degrees of freedom on the normal direction of the stop plate were blocked (Figure 6 and Figure 7).

At the base of the pole, the degrees of freedom are blocked in the radial direction because of the assembly at the base of the pole. 

The software used is Altair Hyperworks. Finite element modeling is performed with the Hypermesh preprocessor, and the analyses are performed with the Optistruct solver, both from the Altair package. 

## 4. Results

Based on the developed model, with the boundary conditions presented above, structure strength calculations are made for several scenarios. The scenarios are developed successively, starting from the initial version proposed by the designers, identifying the areas that present design problems, and trying to improve, successively, the previous solutions.

**Figure 8:**
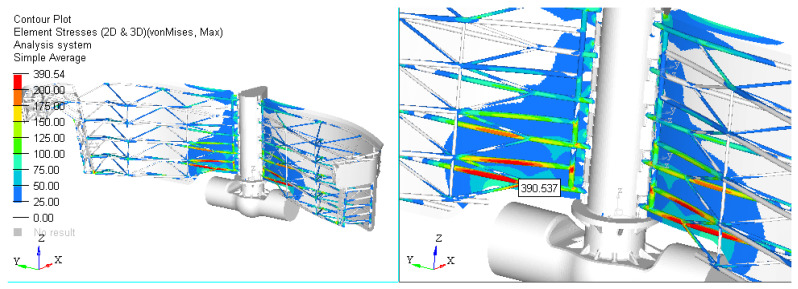
Scenario no. 1: stress field in the dam gates (MPa) for Case 1; $p_{1}=0.054\;\mathrm{MPa}$.

**Figure 9:**
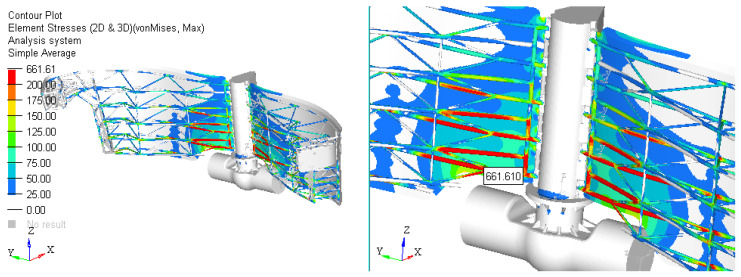
Scenario no. 1: Stress field in the dam gates (MPa) for Case 2; $p_{2}=0.069\;\mathrm{MPa}$.

Scenario 1. The first results are presented for the initial solution proposed by the designer. With the chosen constructive solution, the calculations are performed using FEM software. Figure 8 shows the gate stress field for Case 1 and Figure 9 shows Case 2.

The finite element analysis performed at this stage takes into account only the hydrostatic phenomena that may occur in the functionality of the gate system.

The material considered in the analysis of the set of gates is steel, with the usual mechanical properties used in finite element analyses, namely: modulus of elasticity E = 210,000 MPa, Poison ratio ν = 0.3, and density *ρ* = 7860 kg/m^3^. The stress limit of the material considered is 200 MPa.

Based on the finite element analysis performed, the following can be concluded:

❑ The maximum stress for both cases analyzed exceeds the stress limit. In this situation, the design must be improved until this criterion is accomplished. The place of occurrence of the maximum stress is on the structure of bars. The values obtained on cases are the following:
  - Case 1: Stress = 390.54 [MPa]
  - Case 2: Stress = 661.61 [MPa].
❑ Solutions proposed to reduce the stress in the assembly:
  - Strengthening the reinforcing bar system by increasing the thickness of the bars or their cross sections.
  - Application of a honeycomb plate system especially in the area close to the pole where the highest displacements obtained stresses in the structure.
  - Use of lighter materials (aluminum alloys or composite) to reduce the weight of the gate assembly (on the current design, a subassembly located on one side or the other of the column has 77.5 tons). By reducing the weight, lower inertias of the system will be obtained, especially for the situations of shock closing of the gates.
  - It is recommended to continue the static analysis scenarios with a dynamic analysis to take into account the average speed of a water stream.

**Figure 10:**
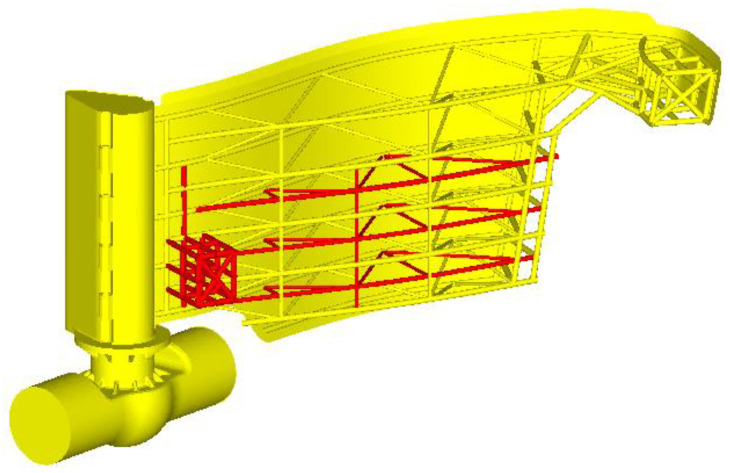
The improved beam structure.

Scenario 2. Compared to the previous analysis, on the current design was added the beams structure and is represented in red in Figure 10.

**Figure 11:**
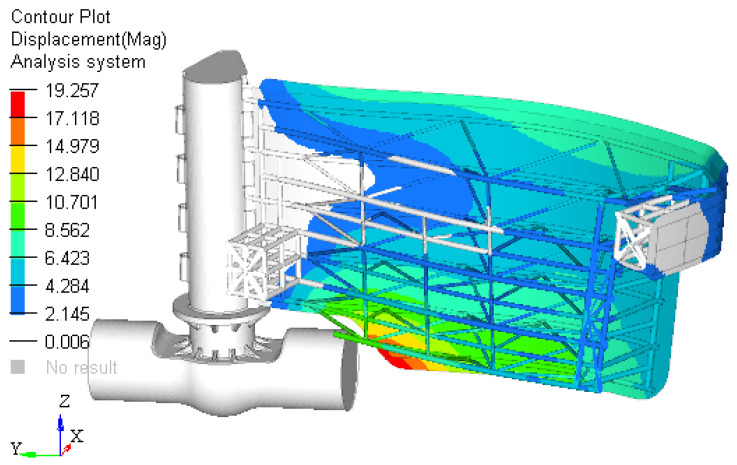
The displacements field for the structure of the gate (mm) for Case 1.

The model is analyzed only on the half because is symmetric and the plot is similar for the other side. The maximum displacement obtained in case 2 is close to 20 mm (Figure 11). Compared to the previous analysis, the value obtain is reduced with 70%. 

**Figure 12:**
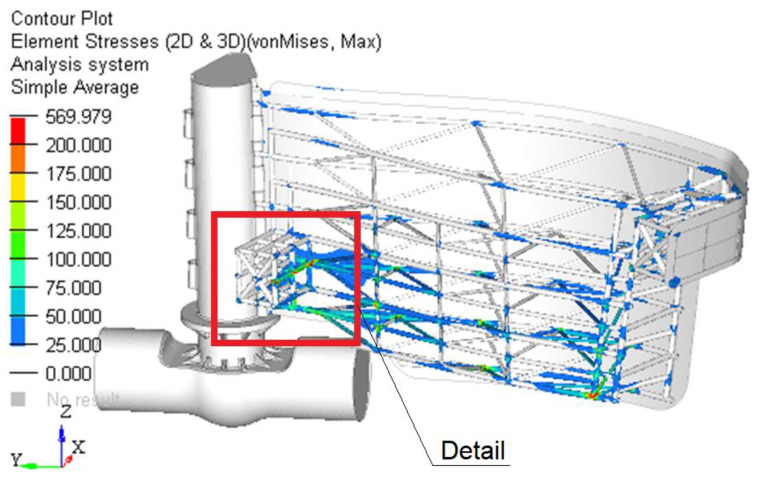
The stress field in the reinforced structure of the gate.

**Figure 13:**
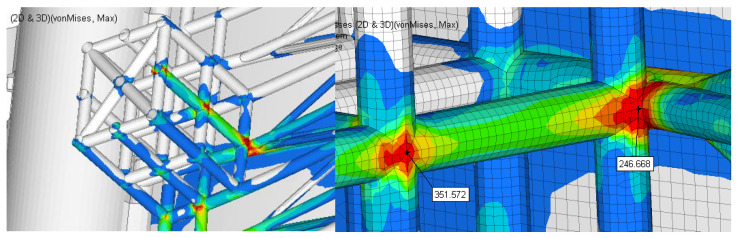
Details of the zone with maximum stress.

Figure 12 presents the field of stress in the structure of gate and Figure 13 shows the details of this field.

The resulting maximum stress is obtained in the beam structure in the area closer to the middle area. The maximum value is approximately 352 MPa. Compared to the previous solution, the maximum stress is reduced by 53%.

Based on the finite element analysis performed, the following can be concluded:

❑ The maximum resultant stress on the second case analyzed (the worst case) is reduced from 661 MPa to 352 MPa, which is 50%.
❑ The global displacement magnitude is also reduced from 77 mm to 20 mm.

A way to improve the solution suggested by the previous remarks can be as follows:

- Strengthening the reinforcing bar system by increasing the thickness of the bars or their cross sections, especially for the bars that have a level of stress that exceeds the yield.
- Application of a honeycomb plate system especially in the area close to the pole to increase the stiffness and reduce the global displacement.

**Figure 14:**
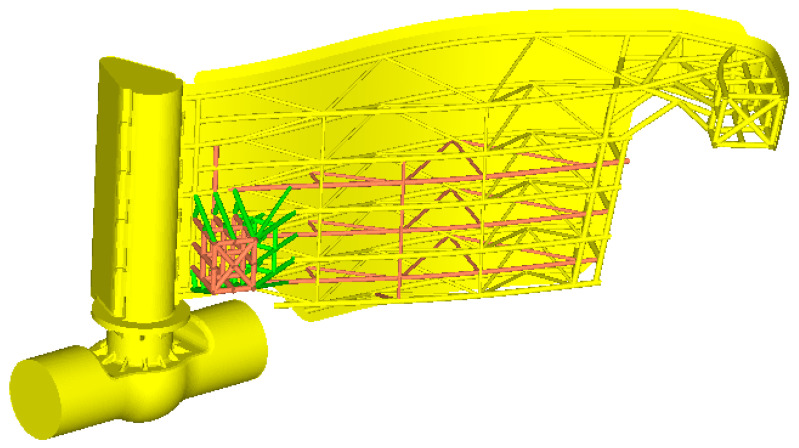
The beams structure for the gate in the improved Scenario no. 3.

Scenario 3. To the structure analyzed in the Scenario 2, more beams in the structure are added, as represented in green in Figure 14.

**Figure 15:**
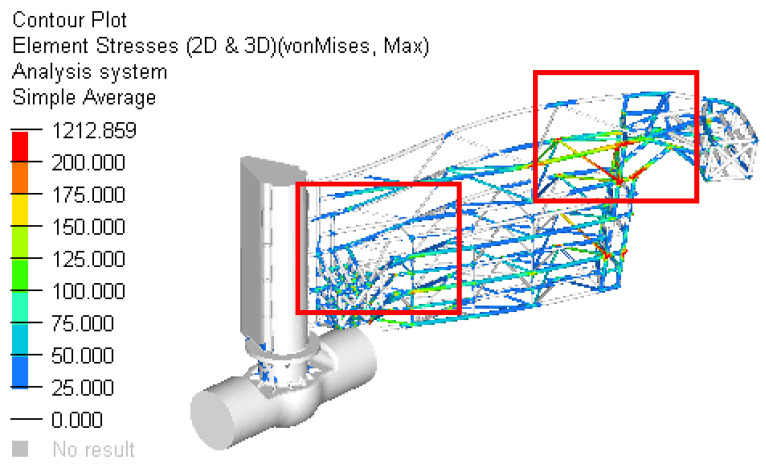
The stress field in the reinforced structure of gate for Case 1.

**Figure 16:**
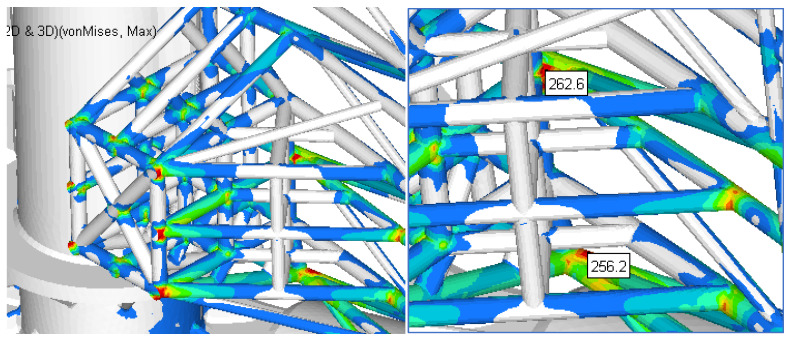
Details of the zone with maximum stress for Case 1.

**Figure 17:**
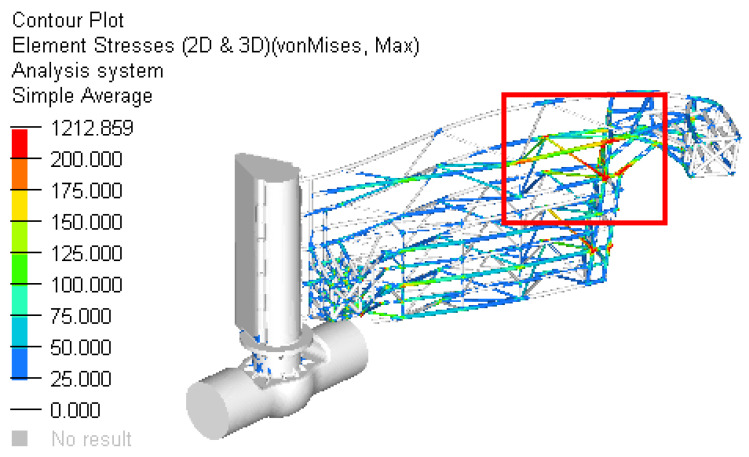
The stress field in the reinforced structure of gate for Case 2.

**Figure 18:**
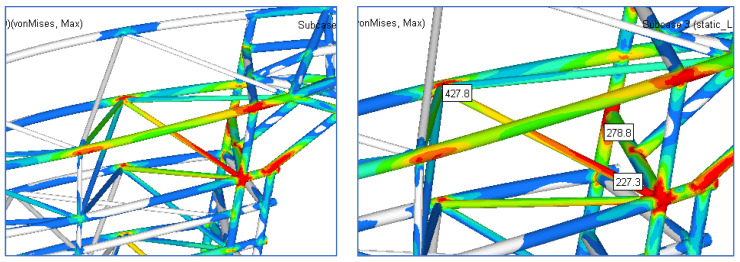
Details of the zone with maximum stress for Case 2.

The following figures present the fields of stresses for the two cases (Figure 15, Figure 16, Figure 17 and Figure 18).

Figure 15 presents the field of stress in the structure of the gate for Case 1 and Figure 16 shows the details of this field.

Figure 17 presents the field of stress in the structure of gate for Case 2 and Figure 18 shows the details of this field.

**Figure 19:**
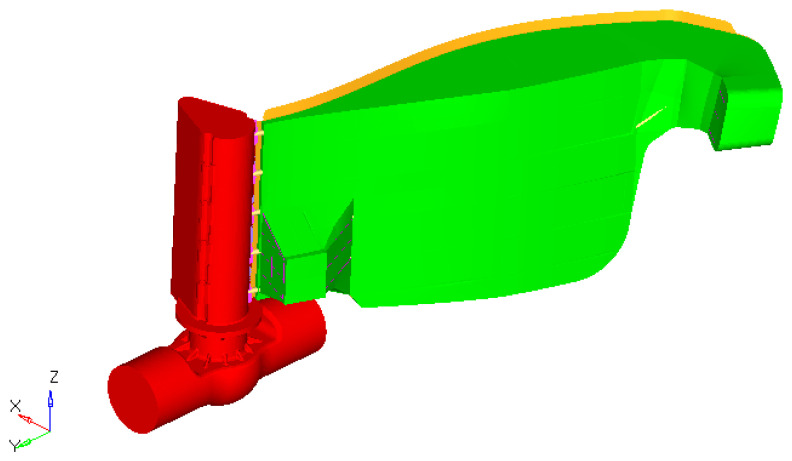
The innovative structure of the gate.

Scenario 4. On the current design, a floating box structure was added to reduce the stress in the bars and also to diminish the weight of the dam (Figure 19).

**Figure 20:**
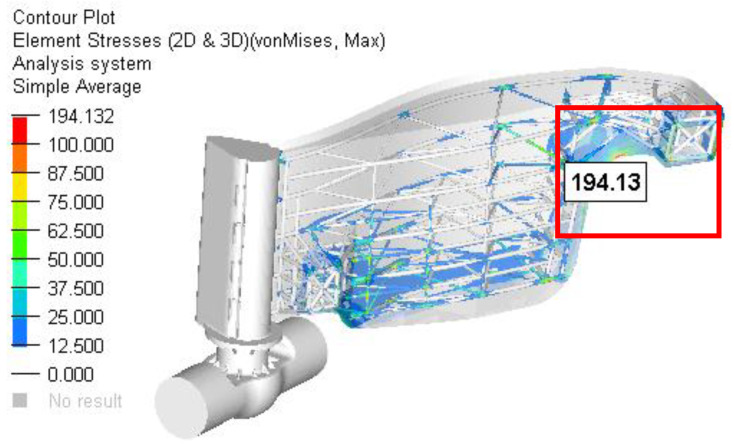
The stress field in the reinforced structure of gate for Case 2.

**Figure 21:**
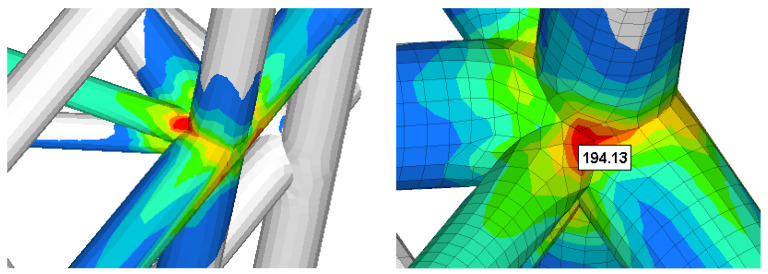
Details of the zone with maximum stress for Case 2.

The resulting maximum stress was obtained in the bars structure situated in the area closer to the outside stop areas (Figure 20 and Figure 21). The maximum value (not influenced by the rigid constraints) was approximately 194 MPa. Compared to the better previous solution, the local maximum stress (in the indicated area) was reduced by 26% (from 262 MPa to 194 MPa).

## 5. Conclusions

Based on all of the previous finite element analyses performed, the following can be concluded:

❑ The principal target was to minimize the maximum stress in the gate structure as much as possible using some of the hypothetical scenarios considered and described above.
❑ The local maximum resultant stress close to the pole zone, in the second case analyzed (the worst case), was reduced from 352 Mpa to 263 Mpa, which is 25%.
❑ The maximum stress moved to the other zone faraway from the polem and the maximum value was 426.7 MPa.

Other solutions to reduce the stress level are as follows:

- Strengthening the reinforcing bar system by increasing the thickness of the bars or their cross sections, especially for the bars that have a level of stress that exceed the yield.
- In the zone with max stress, some additional short bars with a reinforcement role in the local zone can be applied.

The dam model studied in this paper is a new constructive solution in which the gates are closed automatically, when the downstream water level exceeds a certain height. Such a solution does not require auxiliary mechanical systems and a system control. Closing and opening is controlled by the water level. The proposed solution is based on the existence of a central pivot and the effect of the Archimedean forces that cause a swing of the gates. As a result, the experience of the designers in creating such a structure is practically non-existent. Starting from the previous experience regarding the classic dams, a solution for the construction of the dam was proposed (Scenario 1). It was found, following the resistance calculation, that this solution was unsuitable. As a result, three more project scenarios were proposed and it was found that only the last of them provided the necessary strength for operation. The experience resulting from these simulations can be useful to the designers of such dams.

## Data Availability

Not applicable.
